# Breast, Prostate, and Colorectal Cancer Survivors’ Experiences of Using Publicly Available Physical Activity Mobile Apps: Qualitative Study

**DOI:** 10.2196/10918

**Published:** 2019-01-04

**Authors:** Anna L Roberts, Henry WW Potts, Dimitrios A Koutoukidis, Lee Smith, Abigail Fisher

**Affiliations:** 1 Research Department of Behavioural Science & Health University College London London United Kingdom; 2 Institute of Health Informatics University College London London United Kingdom; 3 Nuffield Department of Primary Care Health Sciences University of Oxford Oxford United Kingdom; 4 National Institute for Health Research Oxford Biomedical Research Centre Oxford United Kingdom; 5 The Cambridge Centre for Sport and Exercise Sciences Department of Life Sciences Anglia Ruskin University Cambridge United Kingdom

**Keywords:** physical activity, health behavior, cancer survivors, mobile apps, mHealth, digital health

## Abstract

**Background:**

Physical activity (PA) can improve a range of outcomes following a cancer diagnosis. These include an improvement in experience of side effects of treatment (eg, fatigue) and management of comorbid conditions. PA might also increase survival and reduce recurrence. Digital interventions have shown potential for PA promotion among cancer survivors, but most in a previous review were Web-based, and few studies used mobile apps. There are many PA apps available for general public use, but it is unclear whether these are suitable as a PA intervention after a cancer diagnosis.

**Objective:**

This study sought posttreatment nonmetastatic breast, prostate, and colorectal cancer survivors’ opinions of using smartphone apps to promote PA and gathered their views on existing publicly available PA apps to inform a future intervention.

**Methods:**

Each participant was randomly assigned to download 2 of 4 apps (Human, The Walk, The Johnson & Johnson Official 7 Minute Workout, and Gorilla Workout). Participants used each app for 1 week consecutively. In-depth semistructured telephone interviews were then conducted to understand participants’ experiences of using the apps and how app-based PA interventions could be developed for cancer survivors. The interviews were analyzed using thematic analysis.

**Results:**

Thirty-two participants took part: 50% (16/32) had prostate cancer, 25% (8/32) had breast cancer, and 25% (8/32) had colorectal cancer. Three core themes were identified. The first theme was that multiple factors affect engagement with PA apps and this is highly personalized. Factors affecting engagement included participants’ perceptions of (1) the advantages and disadvantages of using apps to support PA, (2) the relevance of the app to the user (eg, in terms of cancer-related factors, their PA goals, the difficulty level of the app, the way in which they interact with their mobile phone, and the extent to which the app fits with their self-identity), (3) the quality of the app (eg, usability, accuracy, quality of production, and scientific evidence-base), and (4) the behavior change techniques used to promote PA. In the second theme, participants recommended that apps that promote walking are most appealing, as walking removes many barriers to PA. Finally, the participants suggested that PA apps should be integrated into cancer care, as they valued guidance and recommendations from health care professionals.

**Conclusions:**

This sample of breast, prostate, and colorectal cancer survivors was receptive to the use of apps to promote PA. Although no publicly available PA app was deemed wholly suitable, many suggestions for adaptation and intervention development were provided. The results can inform the development of an app-based PA intervention for cancer survivors. They also highlight the wide-ranging and dynamic influences on engagement with digital interventions, which can be applied to other evaluations of mobile health products in other health conditions and other health behaviors.

## Introduction

The number of people diagnosed with cancer continues to increase and is estimated to reach more than 20 million new cases per year worldwide by 2025 [1]. Earlier diagnosis and improvements in treatment mean that rates of cancer survival are also increasing. However, fatigue [2], pain [3], sleep problems [4], weight gain [5,6], and anxiety and depression [7,8] are common among cancer survivors, and 70% have a comorbid chronic condition [9]. Depending on cancer type and the area at which treatment is targeted, particular groups of cancer survivors are at greater risk of more specific late effects. For instance, lymphedema is common among breast cancer survivors [10], and incontinence is common among prostate and colorectal cancer survivors [11,12]. These sequelae can have a profound negative impact on quality of life (QoL) [13], and interventions to improve outcomes in cancer survivors are urgently required.

There is now strong evidence that physical activity (PA) can improve a range of important cancer outcomes for breast, prostate, and colorectal cancer survivors, 3 of the most prevalent cancer types worldwide [1]. Observational evidence shows that PA might reduce cancer-specific and all-cause mortality and cancer recurrence in breast, prostate, and colorectal cancer survivors [14-16]. PA has also been shown to improve overall health-related QoL, emotional well-being, and social functioning and reduce anxiety, fatigue, pain, and sleep problems in cancer survivors [17]. Therefore, cancer survivors are now advised to meet the same PA guidelines as the general adult population. This includes a minimum of 150 min of at least moderate-intensity PA and 2 instances of strength and resistance-based training per week [18-21]. Where this is not achievable, avoiding inactivity is recommended. However, when PA is measured objectively using accelerometers, as few as 16% of breast cancer survivors meet PA recommendations, and those with the highest level of comorbidity are the least active [22]. People diagnosed with cancer are less likely to engage in PA than people who have never received a diagnosis [23]. In a sample of 631 breast cancer survivors, the self-reported proportion meeting PA guidelines declined from 34% at 2 years post diagnosis to 21% at 10 years post diagnosis [24]. Side effects of treatment and fear about what type of PA, when, and how to start or increase PA safely are often reported as barriers to PA after a cancer diagnosis [25-27]. As a result, PA interventions for people affected by cancer are required.

Face-to-face interventions are time- and resource-intensive, and accessibility can be limited [28,29]. Increasing ownership of smartphones provides an avenue for scalable digital behavior change interventions, including those that aim to increase PA. In the context of cancer, for which age is the strongest risk factor [30], smartphone ownership has, in recent years, risen most rapidly among the older age groups. In the United Kingdom specifically, smartphone ownership increased from 32% to 47% in people aged over 55 years from 2015 to 2017 [31,32]. In the United States, smartphone ownership is even higher among older age groups (73% among those aged 50-64 years and 46% in people aged over 65 years) [33].

Digital behavior change interventions have been shown to increase PA in the general adult population [34] and our recent meta-analysis of 15 studies showed that digital interventions have the potential to increase cancer survivors’ moderate-vigorous PA by approximately 40 min per week [35]. However, of the studies included in this review, the majority used Web-based interventions, and only 2 small feasibility studies evaluated the use of mobile apps in PA promotion [36,37]. Mobile apps have the benefit of being able to deliver behavior change techniques (BCTs) in real time using a device that is usually switched on, usually carried with the person, and often has inbuilt functions to monitor PA and deliver immediate feedback. There are many health and fitness apps aimed at the general population that are currently available on commercial app stores, which might already be appropriate for cancer survivors or could be adapted to increase their suitability. Exploring cancer survivors’ experiences of using different types of existing apps is, therefore, a useful way to understand which types of PA apps might be most appropriate or successful, before making potentially large investments into app or intervention development.

Qualitative research methods provide a rich understanding of people’s experiences, thoughts, and opinions and seeking the perspectives of intended users is a critical element of digital intervention development [38,39]. Robertson et al conducted focus groups with breast, prostate, colorectal, and endometrial cancer survivors where feedback was collected for potential PA app features and messages [40]; however, the feedback provided was hypothetical. We suggest that by allowing participants to actually experience using different types of apps and BCTs over a period of time provides greater ecological validity. Since the publication of the meta-analysis, Short et al conducted an experiential mixed-methods study where 10 cancer survivors were referred to one of 15 existing PA apps, which were used for a 1- to 2-week period [41]. Although this study explored the participants’ experience and preliminary efficacy of the app referral service, it did not explore participants’ opinions of using the apps in detail. We see the value in a deeper understanding of participants’ perceptions of their preferences for and influences on engagement with PA apps. For the purposes of this study, we use a broad, integrative definition of engagement comprising “1) the extent (e.g. amount, frequency, duration, depth) of usage and 2) a subjective experience characterized by attention, interest and affect” [42].

Therefore, the aim of this study was to seek breast, prostate, and colorectal cancer survivors’ opinions of using apps to promote PA and gather their views on existing publicly available PA apps to inform a future intervention.

## Methods

### Mobile Apps

During our initial scoping of the smartphone app stores, no apps that were specifically designed to promote PA among cancer survivors were identified. This is in line with a previous Australian study exploring the use of PA apps among cancer survivors [41]. Therefore, the PA apps considered for this study were identified from apps that were featured in the “Health and Fitness” section of the British Apple App Store (iOS), along with other apps that the study authors were aware of from previous work in digital health and that might have been suitable for this study. The following criteria were considered in deciding which apps might be suitable for the study:

- Content: The apps needed to vary from each other in terms of the type of PA, and their format, features, and BCTs to allow comparison between different types of apps.
- Typicality: Although the apps needed to vary in terms of their content, we also felt that the apps chosen should be typical of the various types of popular PA apps that are available (eg, activity trackers and workout programs).
- Suitability: The apps needed to be suitable for people who have undergone cancer treatment and, therefore, needed to have the flexibility to cater for different levels of fitness and familiarity with PA. Given the target group, apps that catered for low levels of fitness/familiarity with PA, but with an option to increase this if required, were of interest. Each app was reviewed for its suitability for use by breast, prostate, and colorectal cancer survivors by a physiotherapist specializing in oncology.
- Stability: The apps were required to have been launched at least 2 years before the study.
- Availability: The apps needed to be available on both iOS and Android devices.

**Figure 1:**
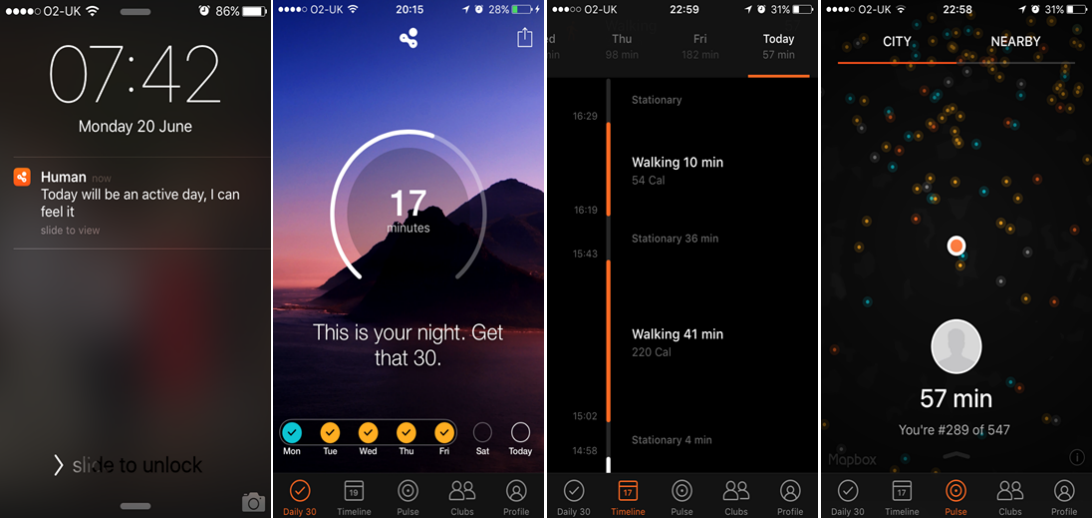
Screenshots of Human.

**Figure 2:**
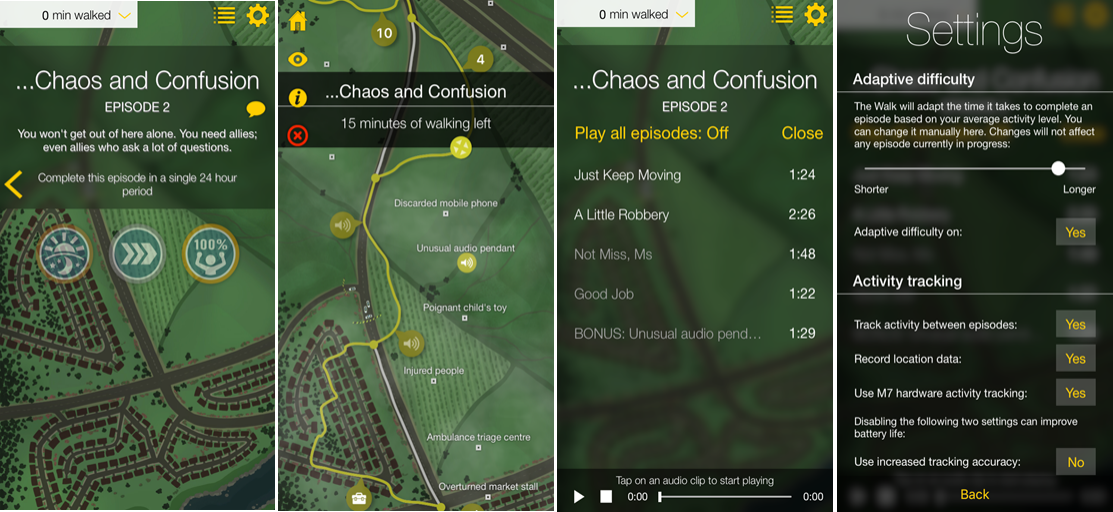
Screenshots of The Walk.

**Figure 3:**
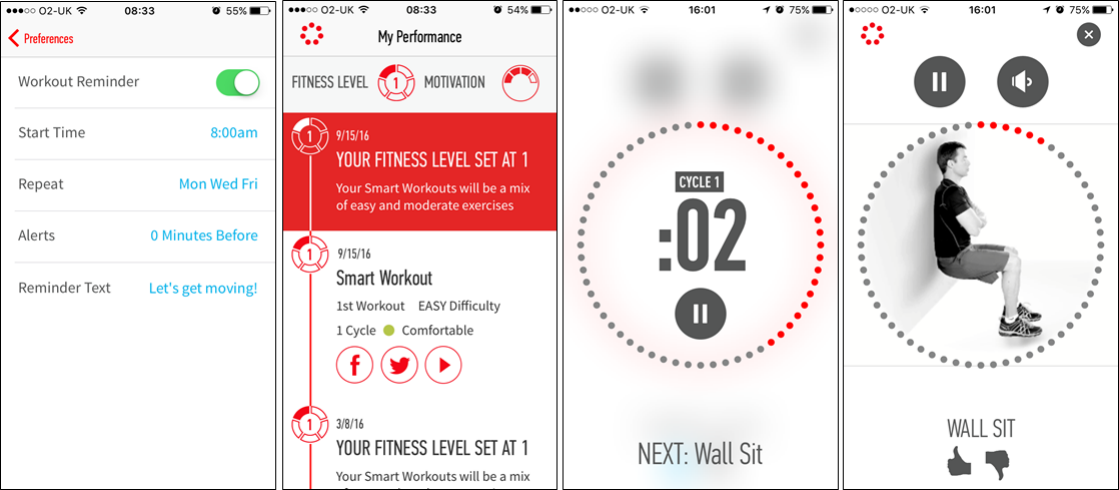
Screenshots of The Johnson & Johnson Official 7 Minute Workout (J&J).

**Figure 4:**
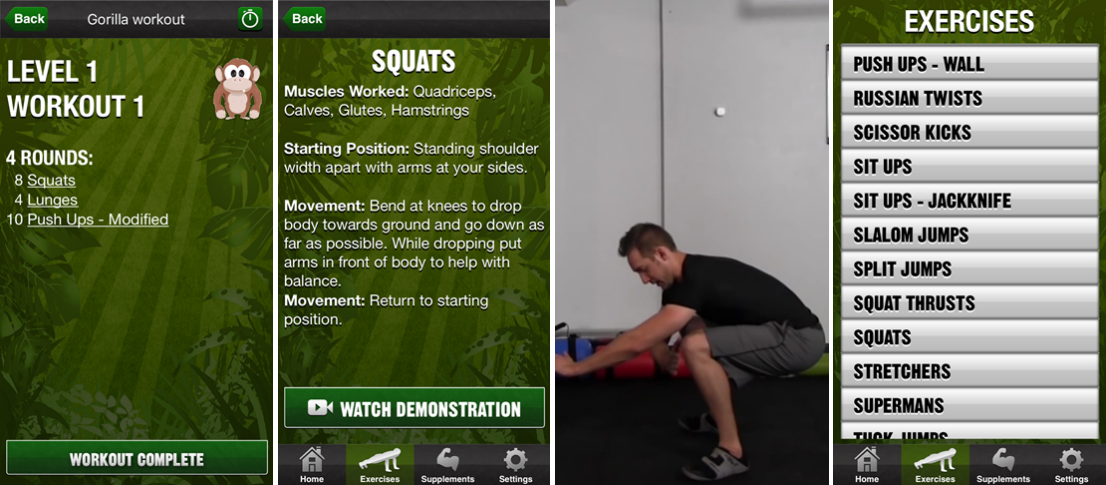
Screenshots of Gorilla Workout.

We felt that 4 apps should be included in the study, based on a number of considerations. These included the number of apps required to compare multiple participants’ opinions across several different PA apps, the number of participants required for the study, and feasibility of recruitment and data analysis. Given the consideration of all of the above factors, the 4 chosen apps were “Human,” “The Walk,” “The Johnson & Johnson Official 7 Minute Workout” (J&J), and “Gorilla Workout” (see Table 1 for a description of each of the apps and an assessment of the incorporated BCTs, coded using the BCT Taxonomy (v1) [43] by AR and DK, with discrepancies resolved via discussion). Figures 1-4 show screenshots of the 4 apps.

### Recruitment

Participants were recruited via advertisements within community-based cancer support groups (either by verbal descriptions from group leaders at meetings or via posters, flyers, and email mailing lists), Facebook cancer support groups, and charitable organizations (eg, Macmillan Cancer Support’s Cancer Voices and Tackle Prostate Cancer). We initially aimed to recruit 32 participants to attempt to ensure sufficient representation from participants diagnosed with each of the 3 cancer types and so that approximately 16 participants would be allocated to use each of the 4 apps throughout the study. If new themes continued to be identified, we would continue recruitment until saturation was achieved.

**Table 1:** App characteristics.

App (Developer)	Price	Description	Behavior change techniques
Human (Humanco, Inc)	Free	Encourages users to meet daily 30/60/90/120 min goal of walking, running, and/or cycling measured using mobile phone’s activity tracker. Delivers push notifications when users have not met their goal or during periods of inactivity. Compares activity levels to other app users nearby	1.1 Goal setting (behavior); 2.2 Feedback on behavior; 2.3 Self-monitoring of behavior; 6.2 Social comparison; 7.1 Prompts/cues; 10.3 Nonspecific reward
The Walk (Six to Start)	£2.29 (iOS); £2.59 (Android)	An interactive story-based game where walking unlocks audio clips to hear the next part to the story and other rewards. Time to complete an episode is based on the users’ current physical activity level and walking is measured using the mobile phone’s activity tracker	2.2 Feedback on behavior; 10.3 Nonspecific reward; 10.6 Nonspecific incentive
The Johnson & Johnson Official 7 Minute Workout (Johnson & Johnson Health and Wellness Solutions, Inc)	Free	7-min workouts are created to include aerobic and resistance exercises alternating between upper and lower body, core, and total body exercises. The workouts can be tailored to the users’ current fitness and motivation levels and are provided with detailed video demonstrations and audio guidance	1.4 Action planning; 2.3 Self-monitoring of behavior; 4.1 Instruction on how to perform behavior; 6.1 Demonstration of the behavior; 7.1 Prompts/cues; 8.7 Graded tasks; 9.1 Credible source
Gorilla Workout (Heckr LLC)	£0.79 (iOS); £0.83 (Android)	The default program is tailored to the users’ current fitness level and gradually increases in difficulty. Each exercise has written guidance with an associated video with visual and audio demonstrations. Users can also choose to complete their own selection of exercises (from a list of 43) with the same written/video demonstrations. Daily push notifications are delivered to remind users to complete their workout	4.1 Instruction on how to perform behavior; 6.1 Demonstration of the behavior; 7.1 Prompts/cues; 8.7 Graded tasks

Participants were required to be aged 18 years or older; to have been diagnosed with breast, prostate, or colorectal cancer; to have finished primary curative treatment (as it is likely that individuals still undergoing primary treatment or with metastatic disease might require additional support and monitoring to be active); to have no known impairment or comorbidity that meant a clinician had advised them not to exercise; and to own a smartphone. Although participants were required to have finished primary curative treatment (surgery, radiotherapy, and chemotherapy), participants still taking maintenance hormone therapy or under active surveillance were eligible. Participants were offered a £10 voucher as an incentive for completion of this study and to reimburse the cost incurred if asked to install an app that was not free to download. Ethical approval for this study was granted by the UCL Research Ethics Committee (reference: 7663/001).

### Procedure

Participants took part in an initial short semistructured telephone questionnaire that confirmed participants’ eligibility and requested details of the participants’ sociodemographic information (age, gender, and ethnicity), cancer diagnosis, and their experience of using digital technologies to support PA. Participants were asked to describe their perceptions of their current participation in PA (eg, what types of PA and how frequently). This was asked as an introductory question to build rapport with the participants at the beginning of the study and to provide context. A Web-based random number generator (Randomizer) was used to allocate 2 apps to each participant to allow comparison of app features and content but to minimize participant burden. Guidance in downloading and installing each app was provided, if required. Participants were asked to spend approximately 2 consecutive weeks using the apps, (approximately 1 week using each) and were able to choose the order in which they used the apps to which they were allocated over the 2-week period. Participants were asked to try to use each app at least three to four times throughout that app’s trial week and record any comments or opinions in log sheets provided. After 2 to 3 weeks, each participant completed an audio-recorded semistructured telephone interview, using the interview schedule (Table 2) as a guide.

### Analysis

Telephone interviews were conducted by AR and transcribed verbatim by an external company. A partly deductive and partly inductive approach to thematic analysis was adopted using the stepped approach described by Braun and Clarke [44]. The deductive approach to thematic analysis involved using the BCT taxonomy [43] as a framework to code any interview data where participants spoke about app features used to promote behavior change. The rest of the data were analyzed using an inductive approach through an iterative reading and rereading of the data. An initial coding framework was developed by AR and revised in collaboration with DK, with discrepancies agreed via discussion. AR applied the final codes that were then incorporated into themes during discussion between all authors. After analysis of these 32 interviews, no new themes were identified and recruitment was concluded. Data analysis was conducted in NVivo 11.

**Table 2:** Semistructured interview guide.

Discussion point	Details
Recap	Confirm which apps participant was asked to download and try
Download/install	Ask about the participants’ ability to find, download, and install each app
First app	Ask participant to start by giving overall opinion of app; Depending on amount of detail provided in overview, ask participant to expand on any points raised in overview, comment on likes/dislikes, comment on specific app features/BCTs^a^ (dependent on allocated app)
Second app	Repeat the steps as given for the first app
Appropriateness for cancer	Discuss how appropriate and relevant each of the apps were for their personal circumstances and as a cancer survivor
Adapting for cancer survivors	Discuss how (if at all) the apps could be adapted for cancer survivors. If so, what adaptations/functions to tailor the app would they make
Interest in an app	Discuss participants’ interest in a PA^b^ app tailored specifically for people who have had cancer
Preferred types of PA	Discuss types of PA that should be promoted to cancer survivors, including intensity, frequency, type of activity, and with relevance to current PA guidelines (ie, 150 min moderate-vigorous PA and 2 sessions of strength and resistance-based exercises per week) and how apps could promote these types of PA (if at all)
Recommendations	Discuss any PA recommendations that were provided to them following cancer diagnosis/treatment and who were they delivered by or where participant looked for them
Intervention communication	Discuss who should direct cancer survivors to a cancer-specific PA app, including when this should be discussed and promoted to patients

^a^BCT: behavior change technique.

^b^PA: physical activity.

## Results

A total of 40 participants began the study, and 32 participants completed telephone interviews. Of those who dropped out, lack of time, family circumstances (eg, bereavement), and not wanting to update their smartphone’s operating system or register credit card details with Google Play were the listed reasons. Of the 32 participants who completed the study, the mean age was 60 years (range 37-78 years; SD 11 years) and the other sample characteristics are displayed in Table 3.

Broadly, the core themes demonstrate that multiple factors affect engagement with PA apps and this is highly personalized, that apps that promote walking are most appealing for cancer survivors, and that PA apps should be integrated into cancer care.

### Multiple Factors Affect Engagement With Physical Activity Apps, and This is Highly Personalized

Key determinants of engagement appeared to be the users’ perceptions of (1) the advantages and disadvantages of using apps to support PA, (2) the relevance of the app, (3) the quality of the app, and (4) the BCTs used to promote PA.

#### Perceived Advantages and Disadvantages of Using Apps to Support Physical Activity

The participants identified a number of advantages of PA apps, which facilitated engagement with the apps. These included the convenience that an app offers in terms of equipment required, cost, and not being required to attend a specific exercise facility:

Especially if you can, y’know, the workouts, like the Gorilla workouts that I’ve looked at so far, they’re all just using your own body, where you don’t need any special equipment, and all the rest of it…and you don’t need to spend £30 a month to join a gym to do it.

Male, aged 68 years, colorectal cancer

You can just choose when you decide to do it – so you can think, “right, I’m gonna do a little workout now”, so y’know, pick your moment, put your phone on and just pick whichever one you want.

Female, aged 52 years, breast cancer

They also commented that apps could be useful in building confidence or self-efficacy for PA and how this can be important in relation to side effects:

I was left with a lot of tummy problems after my treatment. So in a way you would think that doing a workout at home might suit a lot of people because if their confidence is low, either how they feel about their fitness or that they need to be near the loo or whatever, then being at home should be reassuring, shouldn’t it?

Female, aged 47 years, breast cancer

**Table 3:** Sample characteristics (N=32).

Sample characteristics		n (%)
**Gender**		
	Female	10 (31)
	Male	22 (69)
**Ethnicity**		
	White British	28 (88)
	White-other	1 (3)
	Asian/Asian British	2 (6)
	Mixed	1 (3)
**Cancer type**		
	Breast	8 (25)
	Prostate	16 (50)
	Colorectal	8 (25)
**Experience of using digital technology to support PA^a^**		
	Never used	10 (31)
	Mobile phone installed PA app (eg, Apple Health and SHealth)	5 (16)
	Currently using a PA tracker (eg, pedometer, Fitbit, Garmen, and Strava)	9 (28)
	Have used a PA tracker before but not currently using	5 (16)
	Using combination of technologies (eg, mobile phone installed PA app + PA tracker)	3 (9)

^a^PA: physical activity.

It was also acknowledged that an app-based PA program could be more effective in comparison with printed materials due to the ubiquity of smartphones and the more engaging nature of interacting with the program in real time:

Where apps, of course, have a huge advantage, the days of paper things…exercise sheets, and things which end up in the bottom drawer or in the dustbin, err, apps are better than that, because they’re on your phone, and they can be updated, as well...you’ve always got your phone with you. You haven’t always got the list with you.

Male, aged 69 years, prostate cancer

...it’s a bit more interactive and it’s there and you can just...I’m gonna press on...whatever this...what’s a box jump? For example, and you can press on that and see...see what it is, so it’s very, very useful.

Male, aged 69 years, prostate cancer

Although only 1 participant mentioned the possible benefit of apps in terms of the level of literacy required to interact with the program, it is important to note that this could improve accessibility to a PA intervention through the visual and interactive features of the apps:

Y’know...it’s a nice, simple app. You don’t need to be that literate.

Male, aged 60 years, prostate cancer

However, a number of disadvantages of app-based PA interventions were also raised. These included the possible safety implications of unsupervised PA:

...if somebody isn’t getting advice from a professional first and they’re just picking up an app and...wanted to get a bit more active and doing it at home, I think that something like this could be actually be quite risky.

Female, aged 43 years, breast cancer

I think you’d have to be careful that people did it properly and that they did it at the right time and didn’t…you know, didn’t overdo it...some people think, ‘ooh, well I’m doing exercise, it must be doing me good,’ but it might not be…cause they’re doing it too early, or they’re doing it wrong...Because there’s no supervision, there’s no guarantee, is there?...That would be more for strength-based things, really [compared to walking].

Female, aged 59 years, breast cancer

Participants also experienced a number of technical issues (eg, impact on battery life, mobile data usage, and smartphone memory):

[Human] does drain your battery quite quickly because you have to use, erm, location services all the time...if it was gonna be a regular thing I wouldn’t use it every day then just because the fact that it does drain your battery.

Female, aged 37 years, colorectal cancer

There were also concerns around data security and access to or usage of personal data:

...of course, with the freebies, as we know, what you’re doing is you’re signing up to allow them to track your location, other things you might be doing...nothing’s really free.

Male, aged 69 years, prostate cancer

#### Perceived Relevance of the App

The participants described a number of factors that influenced their opinions of the perceived relevance of the apps used in this study. The participants described greater engagement with the apps that were perceived as most relevant to them. In relation to cancer, the participants acknowledged that they were a heterogeneous group who will differ in terms of their PA ability and that a successful app must be able to be tailored for this diversity to ensure it feels relevant to the user’s ability:

Everybody who’s had cancer will have a different level of fitness anyway even after cancer, and they’ll have a different level of motivation and a different starting point so that’s why that 7 minute app is good...you can choose...depending on where your starting point is.

Female, aged 52 years, breast cancer

The participants also highlighted that each individual’s experience of cancer, treatment, and side effects differs and that a PA app to be used by cancer survivors must acknowledge the potential barriers that patients who have been diagnosed with various types of cancer and experienced different types of treatment and side effects might experience:

...depending on what treatment you’ve had, in terms of, umm, certainly operations, and scars and whether you’ve got adhesions or...weakened muscles in various places...it’s all going to vary, from one cancer to another...there’s a lot of variation and, err, that needs to be covered.

Male, aged 68 years, colorectal cancer

[Gorilla Workout] came up with something like….I can’t remember what it said, but something like, “Don’t be a slacker, get… you know, get working,” or something, and I was like, “Err…hang on a minute.” Like, if I’m feeling crap and I’m feeling fatigued, that is not what I want to see.

Female, aged 38 years, breast cancer

Furthermore, the participants also described that the types of PA that might feel appropriate or relevant to a cancer survivor could vary depending on where the patient is in their cancer *journey* (eg, diagnosis, treatment, recovery, and survivorship):

I had prostate cancer, and I had an operation. And, if you’re looking at an app to try and get patients who’ve had cancer, y’know, back and fit again, I’m not sure that these exercises [on J&J and Gorilla Workout] were the right ones. I personally felt, that if I were being... had this been about six years ago [around time of treatment], they were too physical. I needed gentler exercises.

Male, aged 70 years, prostate cancer

However, there were also several noncancer-specific factors that influenced the perceived relevance of the app to the participants. These factors included the extent to which the app(s) aligned with the participants’ PA goals:

I suppose it depends what you’re trying to get out of it and, for me, it’s looking at trying to regain a level of fitness, because I’ve probably lost it over the last four months or so. And I see the Seven Minute Workout as the one that will specifically do that whereas, [Human] is just monitoring what I will tend to do anyway.

Male, aged 65 years, prostate cancer

The extent to which the difficulty level of the app was suitable for the user also affected perceived relevance. This was particularly apparent for the strength- and resistance-based training apps:

[Human] was, as I say, very easy. It doesn’t cause you any difficulties or problems. So I think anybody can use it. You know, it doesn’t really matter how fit you are or how unfit you are, it’s not going to be a problem...[with Gorilla Workout] I found, even on the easy level, that some of the exercises were impossible...Level 1 is you can perform 0-10 push-ups, but they still kind of think you’re gonna be able to do some. It’s, like, I can’t do any. And I don’t think I’m ever gonna be.

Female, aged 43 years, breast cancer

Um, and then [J&J] had things like press-ups and the plank. I mean, I just thought it was a joke, to be honest...I had a go on a couple of different days. Um, but it was, it was just much too difficult...I felt quite demoralised when they were so difficult. But yeah, something that’s, um, you know, much more gentle to build up from, um, I think is quite a nice idea.

Female, aged 47 years, breast cancer

The participants also described that the way they interact with their mobile phone affects the perceived relevance of certain types of PA apps, namely activity trackers that require you to carry the smartphone to measure PA behavior:

[Human] assumes your phone is always on you…mine never is, unless I go out. So, it stays on the hall table...So of course, if it’s left on the hall table, you’re not moving around at all. So it’ll say, “You’re pretty inactive,” y’know, “How about a walk around the block?” and you think, err, I’ve been doing the housework all morning. I’m exhausted.

Male, aged 65 years, prostate cancer

Finally, in terms of the participants’ self-identity and their perception of whether the app fits with this identity affected their opinion of its perceived relevance:

And it is a man, isn’t it, doing the exercises?...[J&J] was quite masculine, I think...I know it’s a silly thing but even if it, if there was a choice of having a woman or a man to watch, you know.

Female, aged 47 years, breast cancer

And of course, umm, on both of them [J&J and Gorilla Workout]...the videos, err, show the sort of slim, fit young, ultra-fit, young men doing it. You think, “Gosh, I...I haven’t looked like that for about 40 years.”

Male, aged 69 years, prostate cancer

#### Perceived Quality of the App

The participants described several factors that affected their perceived quality of the apps to promote PA. The participants expressed greater engagement with the apps that were perceived to be higher quality, although they did not necessarily agree which those apps were. The factors affecting perceived quality differed between users.

Primarily, the users described the importance of ensuring that an app is easy to use and intuitive to foster engagement from the first usage:

...the bottom line is that...[The Walk’s] not intuitive...Perhaps I should have looked for a help area, or something, if I wanted to make full use of it, but then I also think, if an app is gonna be good, then it, it needs to lend itself to the user...with Human, again, I didn’t look out for any help areas. It’s just, you start using it, it tells you what, what’s going on, what you’ve done, and you can interpret it quite easily.

Male, aged 51 years, prostate cancer

The participants described the importance of ensuring that an app, which tracks PA behavior, does so accurately:

...the main issue I had was that [Human] would record activity, but it would get it wrong. So when I was out on a bike ride, umm, it had me doing a mixture of walking, cycling, umm or running...so I just felt that it didn’t really work that well for me.

Male, aged 68 years, colorectal cancer

Furthermore, the participants’ description of how well produced the app was affected their perceived quality of the apps:

I kept getting a bit confused with the voices. They weren’t different enough in the story. Mainly, as I say, because, um, it was a bit frenetic and people were noisy and speaking quickly and it was a bit jumpy...and just the production of [The Walk], you know...it was a bit jumbled and thrown together almost.

Female, aged 65 years, breast cancer

The J&J app provided an explanation of the scientific evidence-base behind the recommended exercises and workout program, and this was described as increasing the perceived quality and credibility of the app to benefit health:

I did like the mass of support documentation you could delve down into to find out why the exercises were what they were, and the, umm, sort of, a bit of medical stuff behind it…I felt [J&J] was more medical-oriented…it was looking at your total body, total welfare – and I thought that it felt very professional…I felt the regime was based on good scientific basis.

Male, aged 70 years, prostate cancer

#### Opinions of Behavior Change Techniques Used to Promote Physical Activity

Opinions of BCTs used to promote PA within the apps were sought during the interviews and grouped into the following categories: “video demonstrations;” “prompts/cues (reminders);” “goal setting, self-monitoring, and feedback on behavior;” and “incentives, rewards, and gamification.” Participants’ views toward each of these strategies varied considerably, and their opinions on these BCTs determined the extent to which the participants engaged with the apps to which they were allocated.

##### Video Demonstrations

The use of video demonstrations to illustrate how to perform specific exercises correctly was well received:

...the method of presentation, brilliant. [J&J] was very clear...the bloke was there doing it with you...because you can sort of follow along, without just trying to remember how you should be doing it, and you can look at him to see how he’s got his legs, straight or bent a bit.

Male, aged 51 years, colorectal cancer

##### Prompts/Cues (Reminders)

There was mixed feedback on the use of push notifications/reminders to prompt users to engage in PA and how effective they were. This depended on the users’ opinion on reminders, their tone, and how appropriate they were in terms of the time or context in which they were delivered:

...mixed feelings about the sort of constant reminders [Human] gave you...it’s quite good in some respects, because it does make you think, “Oh, yeah. Okay. I’ll just go and have a quick walk to the end of the road and back.” Err...But then when...three or four are coming, you’re thinking, “Oh god, would you shut up?”...I didn’t mind “Oh, what about a quick walk after lunch?” that sort of thing...they were quite positive.

Female, aged 65 years, breast cancer

...there was at least one of those prompts on Human, that actually we followed it. It said something like, “Let’s go for a walk,” and we said, “do you know what? Let’s do that”...on other occasions, er, we said, well, actually, it’s dark so we’re not...you tend to start ignoring it ‘cause it might not be appropriate at that time...so it wasn’t a bad thing – but it wasn’t always the right thing at the right time.

Male, aged 65 years, prostate cancer

##### Goal-Setting, Self-Monitoring, and Feedback on Behavior

These BCTs were grouped as they are frequently used alongside each other to promote PA. For instance, the Human app presents the daily 30-min PA goal, facilitates self-monitoring of progress toward the goal by presenting data collected by the smartphone’s activity tracker, and then presents feedback on their behavior to indicate whether that goal was met or not. Therefore, it is difficult to separate out the participants’ opinions of each of these BCTs individually; however, the participants generally responded positively to this approach to promote PA:

[Human] does show you like summaries and averages. It gives you some interesting information so you can see whether you’re doing better or worse than you were doing yesterday and that kind of thing...it’s nice to have a target and a challenge to work on.

Female, aged 43 years, breast cancer

I could see that I was actually walking more than I thought. So it all adds up...I think it is interesting to monitor because you can actually see how much you’re doing, and...how quickly actually you reach your target. So you could think, like, “Oh, instead of half hour walking, maybe I could increase it to 45 minutes” or an hour if you want to push yourself. So I think that’s definitely a benefit to monitor it...for me, just the data it was interesting and nice to see what I’m actually doing, and be more aware, and in that sense actually that...that already motivated me...to walk a bit extra instead of the bus...so in that sense...I did walk more with the app.

Female, aged 54 years, breast cancer

Some participants also discussed their positive experience of these types of BCTs using other digital technologies to support PA before this study:

I’ve just got the [Apple] Health one on my iPhone, which we check the steps every day. So because that’s nicely how many steps you’ve done, how far you’ve done, and that 10,000 steps...we’ve both taken that on-board as a very good target...[which is] good because you could have a look and say, “Oh, crikey. I haven’t done enough today” or “I haven’t done enough this week,” or whatever.

Male, aged 69 years, prostate cancer

I find the, you know, the completion of the steps quite satisfying...if I’ve got to the evening and I’m on, you know, nine thousand and something, I want to make sure I’ve got that to 10,000 if I walk up and down the stairs a few times, and then actually when you go over, you know, you do feel quite pleased with yourself...[Fitbit] would plot how many days you’d done, how many steps and what your average was for the week and what your average was for the month and that was quite rewarding, because you do feel like you are achieving something.

Female, aged 47 years, breast cancer

##### Incentives/Rewards and Gamification

There was mixed feedback on the use of incentives/rewards and gamification to increase engagement with the app and PA. This type of BCT was most prevalent in The Walk; however, participants were generally put off using this app by some of the usability issues mentioned above and the extent to which the app was perceived as relevant to them:

[The Walk’s] trying to show you where, you could possibly take alternate...you could select to do a slightly longer walk, and have the chance of getting more points from other things. Like picking up packages, but I haven’t really looked at that.

Male, aged 60 years, prostate cancer

Many of the participants said they felt that the gaming aspect to the app was inappropriate for them and they did not find it interesting:

I’m not interested in doing that, you know. I mean, even listening to [The Walk], it just got boring...I listened to it as I was walking along and I thought this is not for me really, you know, there was people missing here and people hiding there. I didn’t know what it was talking about really. I’m not into that sort of thing.

Male, aged 71 years, prostate cancer

#### Apps That Promote Walking Are Most Appealing for Cancer Survivors

In acknowledging cancer survivors’ varying needs (above), and incorporating their personal experience of cancer with their experience of using the apps in this study, the participants generally agreed that a walking-based app would be most appealing for cancer survivors. Walking was perceived to be safe, accessible, and achievable for the vast majority of people regardless of their ability, cancer type, treatment type, side effects, or where they are in their cancer journey. They also said that walking was enjoyable, which increased the likelihood that it would be sustainable and consequently effective:

First thing to do when you’re coming back from the surgery, or any kind of treatment, I think walking is probably the safest way to introduce yourself back into [an] exercise routine.

Male, aged 51 years, prostate cancer

I couldn’t use my upper body because of the surgery and then I had the chemo and I just couldn’t go to the classes, so…but what I did do was walking, because I thought even if I can’t do anything else you can always walk...if you really talk about something people can do right after or maybe even during treatment, I think walking is the easiest, the safest and the best way to start.

Female, aged 54 years, breast cancer

However, they did acknowledge the need to ensure that participants are engaging in PA that is of high enough intensity to meet the PA recommendations:

People might be having a 10 minute dawdle round the garden centre and think that they’ve done their exercise...I can see the sort of, the, the challenge with getting the balance, um, between the...it being achievable but also being effective isn’t it?

Female, aged 47 years, breast cancer

Some participants recognized the importance of resistance training:

I think walking is very good, but equally I think it’s overall, y’know, a balanced body strength and, and flexibility’s important. So, I think it’s worth persevering with that approach as well.

Male, aged 68 years, colorectal cancer

However, others reported that they did not enjoy or want to do these types of exercises:

I like the walking better than the exercises...the workouts and that sort of thing...I would hate to get...right into the heavy stuff, er, and tiring myself out, you know, cause we are getting older.

Male, aged 70 years, prostate cancer

I don’t like doing exercise, and yet, as I mean in doing strengthening exercise and that sort of thing to build my muscle up, but I don’t mind walking.

Male, aged 70 years, colorectal cancer

#### Physical Activity Apps Should Be Integrated Into Cancer Care

The participants agreed that routinely discussing PA and being directed toward onward support (including apps) within the cancer care pathway would ensure everyone diagnosed with cancer receives support. The participants discussed who would be best placed to direct them toward a PA app and when and how this should be introduced:

##### Patients Should Be Directed to Physical Activity Apps

Participants said that discussions around PA, including being directed toward resources to support behavior change (apps or otherwise), should be discussed with patients as a routine part of cancer care:

I think...there being some sort of formal introduction to the possibility of doing this, then rather it being sort of left for you to find it by yourself...that’s what your expert’s for.

Male, aged 69 years, prostate cancer

I don’t think a lot of people would bother to go out and look, to see what apps they can find to do exercise. So, I think, if you’re gonna do one, I think you’ve got to encourage somehow, you’ve got to encourage people to say, or to go, “Oh, that looks good. I’ll use that one.”

Male, aged 70 years, prostate cancer

##### Health Care Professionals’ Recommendations Are Valued

There was a general consensus that the medical team, in particular the Clinical Nurse Specialist (CNS), would be best placed to discuss PA and possible interventions with patients. Participants reported feeling that they had built a relationship with their nurse and medical care team over the course of treatment and that they would trust the advice they provided as safe, accurate, and beneficial for their recovery:

The specialist nurses – so you always have a breast care specialist nurse who looks after you and if they started talking about it and telling you it was a good thing to do – I would have, I would have definitely done it...because you develop such a relationship with the specialist nurse who’s in charge of your case.

Female, aged 52 years, breast cancer

The nurses. I was assigned a support nurse...She was very good at giving me advice, and support...If she had said to me, “Look, there’s a jolly good app. You will need some ex...you need to get back into fitness again, you’ve had a big op...have a look at this one.” I’d have taken that.

Male, aged 70 years, prostate cancer

This was also discussed in the context of the fear and uncertainty that is often raised when trying to increase PA post cancer and the potential for inaccurate and potentially unsafe information but that they would trust the medical team and CNS:

I didn’t go to any of the support groups although I think they’re a good idea, because people do get, you know, a lot from them. I do think it’s dodgy if you haven’t got a professional person there, because, as I found just sitting in...in the waiting room, um, you know, people have misconceptions...they’ve got their own ideas about their own treatment and their own health, and um, they start feeding people with, as I say, wrong information and wrong facts...so I was sort of aware that I’d just listen to what [the nurses] told me.

Female, aged 65 years, breast cancer

Some participants discussed the impact that receiving PA recommendations and feedback from trusted health professionals had on their subsequent participation in PA:

I had one of my check-ups with my consultant, and she said it might be a good time to introduce a tiny little bit of gentle exercise...and so from that point I then got a Fitbit and starting doing 10,000 steps a day, and by the next time I saw her I’d lost a stone and, um, she was very pleased really.

Female, aged 47 years, breast cancer

Other participants acknowledged that people seek information from different sources, in different ways, so having the information and direction toward an app available via a range of channels might be beneficial:

I think if you want to promote an app like this, it’s, er, it’s a good idea maybe to go, er, yeah, do it via various channels, so both a Clinical Nurse Specialist, er, the oncology physios, or charities, like, er, like Prostate Cancer UK or Breast Cancer Care.

Female, 54 years, breast cancer

##### Physical Activity Should Be Recommended Before and After Treatment

Participants suggested that PA interventions should be discussed at diagnosis or before treatment as a way to help manage or reduce side effects during treatment and after treatment to promote recovery and self-management:

I think if it...if it came as part of the pre-treatment package then I think that would be fantastic, ‘cause you’re already kind of…yes, you’re in a state of shock, but if you’re being given stuff to help and start playing with it before you actually start your treatment...because once you’re in it, it’s quite hard...and then another option, definitely after you finish treatment. Like, if you’re feeling fatigued around radiotherapy time or after, definitely then.

Female, aged 38 years, breast cancer

What I’ve been trialling out [Human] that should be in your initial pack. So you...once you’re diagnosed with the cancer, then you’re given the pack and everything else, what to expect and go through, and I think it should be at that stage, as early as possible...that’s the time you need that information.

Male, aged 54 years, colorectal cancer

## Discussion

### Principal Findings

The sample of breast, prostate, and colorectal cancer survivors interviewed in this qualitative study was receptive to the idea of apps to increase PA but highlighted that it is important to acknowledge the varying needs and preferences of this heterogeneous group. Participants recognized that the impact of cancer on each individual in terms of cancer type, treatment, prognosis, and experience of side effects can be very different, and successful app-based PA interventions must account for that diversity. The results demonstrate the subjective and dynamic nature of engagement with digital interventions and revealed factors that affected engagement for each individual (eg, their perceptions of the advantages and disadvantages of using apps to promote PA, relevance of the app, the quality of the app, and of the BCTs used to promote PA).

Participants recommended that walking would be the most appealing form of PA to recommend using an app and could be recommended at any stage across the cancer trajectory. This was because it was described as feeling safe, achievable, accessible, and enjoyable, regardless of cancer type, treatments received, or ability and could be used to increase confidence and fitness before incorporating strength/resistance-based training as recovery progresses. In terms of the strength/resistance-based training apps in this study (J&J and Gorilla Workout), there was a perception that even the beginner levels of these apps were too difficult and potentially unsafe, given the age, fitness level of many of the participants, in addition to their experience of side effects and recovery from cancer treatment. However, the participants were receptive to the format of these types of apps, with detailed video demonstrations illustrating how to perform each exercise. Activity tracking/walking-based apps did not provoke the same level of unease and the participants said that they felt that these need not be tailored specifically toward people who have had cancer. Although most participants recognized the benefit of strength- and resistance-based training, there was a consensus that apps that promote this type of PA would need to be tailored more specifically toward specific cancer types (eg, with regard to location of surgery) and for people with a lower starting level of ability, confidence, and familiarity with these types of exercises. Some participants also described strength and resistance training as unenjoyable and that they would be unlikely to adhere to these types of regimes. This illustrates the need to increase awareness about other ways of incorporating the strength and resistance training element of the PA recommendations in a way that is more enjoyable or feasible and might be more appealing to this group (eg, yoga, carrying shopping bags) compared with specific workout routines.

The participants suggested that to effectively direct cancer survivors toward an app-based PA intervention, this should be integrated within the existing cancer care pathway and recommended by their health care professionals, particularly CNSs. They described being directed toward an app within the medical setting as providing an opportunity to increase knowledge about the cancer-specific benefits of PA from a trusted source. The participants recommended that discussing PA/directing to ongoing support would be most beneficial before or after treatment, and particularly if it was highlighted as a way to alleviate side effects and promote recovery. They also felt that recommending walking specifically would be appropriate at any point after diagnosis for the majority of cancer survivors.

There is ongoing debate about the most appropriate, feasible, and effective way to support cancer survivors to increase PA within routine cancer care [45-49]. The results of our study support the use of existing PA apps to support low-risk moderate intensity PA (eg, walking) that could help cancer survivors to achieve the recommended minimum of 150 min of at least moderate-intensity PA per week [18-21]. However, one of the main issues of concern for the participants in this study was the lack of supervision and the potential for harm, particularly regarding the resistance training apps, especially for patients who are unfamiliar with these types of exercises or who might require specialist support. Although patients might receive more appropriate and tailored support if delivered and supervised by appropriate allied health professionals (eg, clinical exercise physiologists and physiotherapists) in specialist facilities [48] where adherence to the regimen can be monitored, there are issues regarding access and uptake [50]. A recent UK study found that despite national guidelines recommending that prostate cancer survivors treated with androgen deprivation therapy should receive 12 weeks of supervised exercise training, only 17% of National Health Service (NHS) trusts are able to provide this [51]. This reflects the lack of availability of these programs and the difficulty of implementation in routine care, particularly if uptake is poor. Future work should aim to better understand the potential for apps to support PA, which is likely to require greater involvement and supervision from exercise oncology specialists (eg, resistance training) and with greater adaptation/tailoring based on the individual’s type of cancer, experience of treatment (eg, surgery, hormone therapy, chemotherapy, or radiotherapy) and associated consequences of treatment and side effects (eg, stoma, cachexia, or lymphedema). Greater supervision is also likely to be required for people with advanced/metastatic disease.

However, as highlighted by the participants in this study, there is little debate about the value that patients place on the recommendations provided by their clinical team, particularly the CNS and consultants [51-53]. Despite this, few cancer survivors receive PA recommendations or referrals to exercise programs within routine care [51,54]; health professionals report little discussion about PA with their patients and low awareness of PA recommendations for cancer survivors [52,55-57]. Therefore, it is crucial that oncology staff are supported to have discussions about PA with patients, direct them toward behavioral support to increase PA, and refer to specialist programs, where available. The implementation of recommendations to appropriate PA apps in cancer care requires greater exploration.

Most research in PA and cancer has been overrepresented by female cancer survivors’ and primarily by women who have had breast cancer. For instance, in a meta-analysis exploring the effects of PA after cancer conducted by Fong et al [58], 25 of the 39 included studies were conducted exclusively in breast cancer patients. Although only 6 of the 15 studies included in our review exploring the impact of digital interventions on PA in cancer survivors were conducted exclusively with breast/endometrial cancer survivors, the other 9 studies were all overrepresented by female participants [35]. However, in this study, 69% (22/32) of our sample were male, driven by the 50% of our participants with prostate cancer. It would be interesting to explore the demographic characteristics or particular cancer types for which PA apps are most appealing on a larger scale.

Our approach, enabling participants to experience searching for, downloading, and using selected apps *in the wild* for a period of time, proved to be a time- and resource-efficient method, allowing us to understand how cancer survivors actually experience different types of apps and BCTs. We suggest this provides greater ecological validity than previous studies in the area that have, for instance, sought feedback of hypothetical app features and example text messages from slideshows shown to focus groups of cancer survivors [40]. Digital health research has come to appreciate the importance of usability, design, and tailoring for engagement [38,59]; however, recent reviews have conceptualized engagement with digital health interventions more broadly [42,60]. These reviews have highlighted factors such as personal agency and motivation, personal life and values, the engagement and recruitment approach, and the quality of the digital health intervention [60] and the delivery method (eg, aesthetics/design, ease of use, personalization, and message tone), content (eg, BCTs such as feedback and reminders), the population (eg, demographic characteristics, personal relevance, and self-efficacy), and both the social (eg, norms and social cues) and physical (eg, health care system, location, and time) settings as being important for engagement [42]. Our methodology has allowed us to demonstrate these broader influences on engagement, and we suggest that this methodology could be useful in the development and evaluation of other mobile health (mHealth) products for other health conditions and other health behaviors.

But, how should we respond to the demand from participants for highly tailored interventions that feel relevant to each individual user? Will it be more appropriate to identify/develop a number of PA apps that are suitable for different groups of cancer survivors and from which they could choose the one they think is most suited to them rather than attempting to develop one app that is flexible enough to meet all needs and preferences of a heterogeneous group of individuals? Should we focus on making apps that are cancer specific, or choosing among existing noncancer-specific apps and focusing on how the app is introduced to the individual? In light of this challenge, Short et al [38] have developed a PA app referral scheme to select the most appropriate publicly available, noncancer-specific PA app for a cancer survivor based on a referral matrix, taking into account the participant’s fitness level, PA interests, app preferences, and personality characteristics [41]. This novel approach to evaluation of multiple PA apps within a referral scheme takes advantage of the large number of appropriate and relevant publicly available PA interventions, while offering flexibility, choice, and tailoring to the users’ needs and preferences.

### Limitations

This study should be viewed in light of a number of limitations. The sample was self-selecting. This led to a high proportion of participants who were already physically active and who were interested in technology and their health and recovery. We did not quantify the participants’ current level of PA; however, none of the participants reported being completely inactive. Although this study intended to explore initial opinions of the use of PA apps among cancer survivors, we need to understand the views of those who are inactive or engaging in very little PA, who might feel less confident in engaging in PA or using apps, and who might be unaware of the benefits of PA postcancer diagnosis. Our approach to recruitment means we cannot estimate the number of eligible people who saw the advertisements versus those who responded. Although the participants in this study were able to use the selected apps for between 2 and 3 weeks, a more realistic experience than discussing hypothetical app features in a single session, this does not completely reflect *real-life* app usage or engagement. Participants did not choose the apps, and we did not assess experiences in the longer term. This might be amplified by the fact the participants knew they were taking part in a research study and so might have been more inclined to persevere with some of the apps they disliked and may have discontinued using otherwise.

### Conclusions

In conclusion, this sample of breast, prostate, and colorectal cancer survivors were receptive to the use of apps to promote PA but felt that for apps to be effective among this group, they must feel relevant to the individual. This includes accounting for the needs of those who have been diagnosed with different types of cancer, experienced different types of treatment and side effects, and have different levels of PA ability. Walking was highlighted as the most appealing type of PA to promote via an app as it is perceived as safe, achievable, accessible, and enjoyable. We suggest it is useful to also consider the impact of the users’ perception of the relevance of an app and how an app relates to their self-identity. This can arise from the app features, but might also be affected by how the app is introduced (eg, by a trusted health professional). Digital health research has come to appreciate the importance of usability and its impact on engagement. Our methodology has allowed us to demonstrate the broader and more dynamic influences on engagement with apps, and we believe this work could, therefore, generalize to evaluations of mHealth products for other health conditions and other health behaviors.

## Abbreviations

BCT: behavior change technique
CNS: clinical nurse specialist
J&J: the Johnson & Johnson Official 7 Minute Workout
mHealth: mobile health
NHS: National Health Service
NIHR: National Institute for Health Research
PA: physical activity
QoL: quality of life
